# Knowledge Gaps and Systemic Challenges in Antidepressant Prescribing: Insights from Jordanian Psychiatry Practice

**DOI:** 10.3390/healthcare13222954

**Published:** 2025-11-18

**Authors:** Bayan Abdulhaq, Walid Sarhan, Mohammed Saadeh, Seif Alkayid, Dana Tahseen Libzo, Mohammad Sadaqa, Latefa Ali Dardas

**Affiliations:** 1 School for International Training, Brattleboro, VT 05302, USA; bayan.abdulhaq@sit.edu; 2 Independent Researcher, Amman 121, Jordan; 3 School of Medicine, University of Jordan, Amman 11942, Jordan; 4 Department of Community Health Nursing, School of Nursing, University of Jordan, Amman 11942, Jordan

**Keywords:** depression, guideline adherence, psychiatrists, KAPs, LMICs, Jordan, psychopharmacology

## Abstract

**Background**: Antidepressant medications are the cornerstone of depression treatment worldwide, playing a central role in reducing the burden of depressive disorders. Their appropriate use is particularly important in low- and middle-income countries (LMICs), where the prevalence of mental health conditions is high and health systems face increasing demand. Despite the clinical importance of antidepressants, limited evidence exists on how psychiatrists in LMICs prescribe these medications. Jordan, a middle-income country with a growing mental health burden, provides a valuable case study for understanding prescribing patterns and identifying areas for improvement. Objectives: This study examined (1) the knowledge, attitudes, and prescribing practices of Jordanian psychiatrists regarding antidepressant medications, and (2) the perceived challenges hindering optimal prescribing. **Methods**: A mixed-methods design was employed. Quantitatively, a cross-sectional survey was administered to licensed psychiatrists in Jordan (n = 108; response rate 79.4%). The instrument was adapted from previously published tools on psychotropic prescribing practices and refined using international guidelines and recent reviews on antidepressant use. Qualitatively, semi-structured interviews were conducted with a purposive sample of four psychiatrists to explore systemic, clinical, and contextual barriers to antidepressant prescribing. Survey data were analyzed using descriptive statistics and regression analyses, while interview transcripts were subjected to thematic analysis. **Results**: Although psychiatrists demonstrated a reasonable understanding of antidepressant pharmacology, important gaps were evident. Only one-third (34.3%) recognized Ministry of Health (MOH) guidelines, while nearly four in ten (37.4%) felt international guidelines did not fully apply to Jordan’s population. Laboratory monitoring for metabolic side effects was inconsistently applied, with just 17.6% always requesting such tests and 11.1% never doing so. Consultation with internal medicine for patients on multiple medications was not routine, reported as “sometimes” by 69.4% of psychiatrists. Attitudes toward prescribing reflected caution, particularly in managing pregnant or lactating women, where only half (51.0%) supported discontinuation and three-quarters (75.9%) preferred dose or drug adjustment. Early-career psychiatrists showed lower engagement, as knowledge and attitude scores were significantly higher among those with 11–20 years of experience compared to those with ≤10 years (*p* < 0.001). Overall, the findings highlight uneven application of evidence-based practices, reliance on personal clinical judgment, and limited engagement with national standards. **Conclusions**: Although safety and patient outcomes are valued, systemic, clinician, and patient-related barriers constrain optimal practice. Strengthening national guideline dissemination, ensuring medication access, and supporting continuing professional development could improve prescribing practices in Jordan and similar LMIC contexts.

## 1. Introduction

Mental disorders represent a substantial and growing public health challenge in low- and middle-income countries (LMICs), where limited resources, workforce shortages, and fragile health systems hinder the delivery of effective mental health services. These systemic constraints often coexist with high population needs, conflict, and displacement, compounding the treatment gap. Jordan exemplifies these challenges. According to the Global Burden of Disease study in 2019, the prevalence of mental health conditions in Jordan was estimated at 15.18% [1]. This burden is further exacerbated by an underdeveloped mental health system, characterized by resource limitations, shortages in specialized professionals, and the continuing impact of the regional refugee crisis [2].

Depression is a major global health burden, affecting more than 300 million people worldwide [3]. It is one of the most prevalent mental health conditions in both LMICs and Jordan [1]. Management of depression depends on severity; it is primarily managed through psychotherapy for mild severity and pharmacotherapy for moderate to severe cases [4]. Over the past few decades, several classes of antidepressants have been developed, most targeting monoaminergic pathways. These include selective serotonin reuptake inhibitors (SSRIs) such as sertraline, fluoxetine, and citalopram; serotonin–norepinephrine reuptake inhibitors (SNRIs) like duloxetine and venlafaxine; and tricyclic antidepressants (TCAs) such as amitriptyline and nortriptyline. SSRIs are the most widely prescribed due to their comparatively favorable safety profile, with fewer adverse effects and lower risk of severe toxicity in overdose [5]. Despite their clinical benefits, adherence to antidepressant treatment remains suboptimal, with nearly one-third of patients discontinuing prematurely [6]. Contributing factors include limited patient education, stigma, inadequate follow-up, and concerns about side effects or efficacy [7,8]. Moreover, individual variability in pharmacokinetics and pharmacodynamics, comorbidities, and drug–drug interactions complicate treatment, especially since antidepressants are also prescribed for other conditions, whether psychiatric, such as anxiety disorder, obsessive–compulsive disorder [9], or non-psychiatric, such as neuropathic pain, fibromyalgia, migraines, and insomnia, among others [10].

Optimal prescribing practices are central to achieving effective and safe treatment outcomes in depression care. International guidelines underscore the importance of evidence-based antidepressant prescribing—including appropriate drug selection, dose initiation, titration strategies, and systematic monitoring of patient response—to enhance therapeutic benefit and patient safety [11]. These recommendations are especially critical for vulnerable groups such as the elderly, children, and adolescents, where most dosing regimens are extrapolated from adult studies; as such, low initial dosing and careful follow-up are advised to mitigate risk in these populations [12].

Ensuring optimal prescribing also requires consistent follow-up, awareness of drug–drug interactions, and consideration of patient-specific factors such as comorbidities, weight, and genetic variability in drug metabolism. However, implementing such practices can be especially complex in LMICs, where limited healthcare infrastructure and workforce shortages constrain the availability of specialized psychiatric care. Jordan, for example, has only three mental health hospitals for adults, one specialized psychiatric hospital for children, and 64 outpatient facilities serving the entire population [13,14,15]. The number of trained professionals remains limited, with approximately two psychiatrists, 0.27 psychologists, and 0.04 psychiatric nurses per 100,000 inhabitants [13,15].

Despite the centrality of antidepressants in clinical practice, little is known about the extent to which psychiatrists in Jordan adhere to evidence-based prescribing recommendations, how they approach complex clinical scenarios, and what systemic or contextual barriers influence their decision-making. Understanding these factors is critical to aligning prescribing practices with international standards and improving outcomes for patients with depression.

The purpose of this study was to comprehensively examine antidepressant prescribing among psychiatrists in Jordan, with a particular focus on identifying gaps in knowledge, variations in clinical attitudes, and deviations from evidence-based practice. The study integrated quantitative data on prescribing behaviors with qualitative insights into contextual and systemic barriers to generate an empirically grounded understanding of how antidepressant treatment is approached in Jordan and to inform strategies for optimizing guideline adherence, patient safety, and quality of psychiatric care in similar low- and middle-income settings.

## 2. Methods

### 2.1. Study Design

This study employed a mixed-methods design, combining a cross-sectional survey with in-depth semi-structured interviews. The quantitative component aimed to assess psychiatrists’ knowledge, attitudes, and prescribing practices regarding antidepressant medications. The qualitative component explored perceived challenges and contextual barriers influencing antidepressant prescribing in Jordan. Using a mixed approach allowed for a more comprehensive understanding of both measurable practices and the lived experiences of practitioners. The study received ethical approval from the Institutional Review Board of the University of Jordan.

### 2.2. Setting and Participants

Eligible participants were licensed psychiatrists practicing in Jordan at the time of the study. Psychiatrists who were retired or primarily practicing in non-psychiatric specialties were excluded. Based on the International Medical Corps report, the total number of practicing psychiatrists in Jordan is estimated to be approximately 200 [13]. A priori power analysis was conducted using G*Power version 3.1 to determine the minimum sample size required for the quantitative component. Assuming a medium effect size (f^2^ = 0.15) for multiple regression analysis, an alpha level of 0.05, and statistical power of 0.80, the required sample size for a model with up to six predictors was N = 98. The final analytic sample of 108 psychiatrists exceeded this minimum threshold, ensuring adequate power to detect meaningful associations among knowledge, attitude, and prescribing practice variables. This sample also represents approximately 54% of all licensed psychiatrists currently practicing in Jordan, indicating strong coverage and representativeness of the target population. The qualitative component, involving four in-depth interviews, was guided by the principle of information saturation, which was achieved when no new themes emerged across transcripts.

To secure the needed sample, a list of potentially eligible psychiatrists was obtained from the Jordan Medical Association, following a strategy of national survey of second-generation antipsychotic monitoring in Jordan. Using a convenience sampling method, a total of 136 psychiatrists were approached, and 108 completed the survey (response rate = 79.4%). This sample of 108 respondents represents approximately 54% of the total estimated psychiatrists in the country. For the qualitative strand, purposive sampling was used to select four psychiatrists with varying years of experience, practice settings, and training backgrounds to maximize diversity of perspectives.

### 2.3. Quantitative Survey

The quantitative survey was designed to capture psychiatrists’ knowledge, attitudes, and prescribing practices regarding antidepressant treatment. The instrument was adapted from previously published tools assessing psychotropic prescribing patterns, including a national survey of antipsychotic prescribing in Jordan [16], and further refined using recommendations from international antidepressant treatment guidelines [4,11] as well as recent reviews on antidepressant use in clinical practice [17].

The final questionnaire consisted of four domains:

1. Knowledge of pharmacokinetic and pharmacodynamic principles relevant to antidepressant use.
2. Attitudes toward the utility, feasibility, and safety of evidence-based prescribing in clinical practice.
3. Practices, including prescribing behaviors, dose initiation and titration strategies, follow-up frequency, and laboratory monitoring when indicated.
4. Adherence to international and national antidepressant prescribing guideline recommendations.

Each domain included multiple items scored on Likert-type scales. Knowledge items were based on established pharmacological principles and guideline content, while attitude items assessed agreement with evidence-based recommendations and perceived barriers to their integration. Cronbach’s Alpha for the knowledge and attitudes scales were 0.912 and 0.877, respectively. Practice and adherence items captured self-reported clinical behaviors and the extent of alignment with published standards.

The survey was developed in English, consistent with medical training and clinical documentation in Jordan, and was pilot-tested among a small group of psychiatrists to ensure clarity, cultural appropriateness, and face validity. Feedback from the pilot phase informed minor wording adjustments. Demographic information (age, sex, years of experience, training background, practice setting, and publication record) was also collected. Supplementary Materials File S1 shows the quantitative survey.

### 2.4. Qualitative Interviews

Semi-structured interviews were conducted with four psychiatrists to explore in greater depth the perceived challenges influencing antidepressant prescribing in routine psychiatric practice. The interview guide covered domains such as awareness and use of clinical guidelines, decision-making processes when selecting and adjusting antidepressant treatment, patient-related barriers to adherence, and systemic or infrastructural constraints. Interviews were conducted in English or Arabic depending on participant preference, lasted approximately 30–45 min each, and were audio-recorded with consent. Supplementary Materials File S2 shows the complete interview guide.

### 2.5. Data Analysis

For the quantitative component, data were entered into SPSS (version 28; IBM Corp., Armonk, NY, USA). Descriptive statistics were calculated for demographic and survey items. Reliability of multi-item scales (knowledge, attitudes) was assessed using Cronbach’s alpha. Group comparisons were performed using chi-square tests, *t*-tests, or ANOVA as appropriate, and associations between variables were assessed using logistic regression models. Significance was set at *p* < 0.05. Effect sizes were calculated and interpreted following Cohen’s guidelines to complement significance testing. For group comparisons, Cohen’s d or eta squared (η^2^) were used to quantify the magnitude of mean differences. For regression analyses, R^2^ and adjusted R^2^ were reported as indicators of explained variance. Thresholds of small (η^2^ = 0.01, d = 0.20), medium (η^2^ = 0.06, d = 0.50), and large (η^2^ = 0.14, d = 0.80) effects were applied.

For the qualitative component, audio recordings were transcribed verbatim and translated into English where necessary. Data were analyzed using thematic analysis, following Braun and Clarke’s six-step approach. Two researchers independently coded transcripts, developed initial themes, and then met to refine the coding framework. Discrepancies were resolved through discussion to ensure rigor and dependability. Strategies to enhance trustworthiness included maintaining an audit trail, peer debriefing, and providing thick descriptions of participant responses.

## 3. Results

A total of 108 psychiatrists participated in the study. The majority were male (72.9%), with a mean age of 38 years (SD = 11.68). Most respondents had completed their medical education in Jordan (62.1%), followed by Iraq (9.5%), Algeria (5.3%), and Egypt (5.3%), with smaller proportions trained in Ukraine, Syria, the UK, China, and other countries. In terms of professional experience, over half (52.4%) had between 0 and 5 years of practice, while 21.9% reported 11–20 years, 13.3% had 6–10 years, and 12.4% had more than 20 years of experience. Regarding job position, slightly more than half were consultants (51.0%), while 24.0% were senior residents and 25.0% junior residents. Participants reported diverse work settings, with 36.5% practicing in private clinics, 34.6% working in hospital wards, and 18.3% in public clinics, while a small group worked across both public and private sectors (7.7%).

Most psychiatrists reported working 8–12 h daily (61.0%), and patient loads were fairly evenly distributed, with 41.9% seeing fewer than 10 patients per day and another 41.9% seeing 11–29 patients daily, while 16.2% reported ≥30 patients per day. Monthly income varied, with the largest group earning JOD 501–1000 (40.2%), followed by 24.5% earning JOD 1001–2000 per month. About one-quarter reported a personal psychiatric history (23.1%) or a family psychiatric history (22.1%). In terms of academic activity, 43.3% had no research publications, 41.3% had authored between one and five, and fewer than 16% reported six or more publications. See Table 1.

### 3.1. Knowledge of Antidepressants

While psychiatrists demonstrated a reasonable understanding of antidepressant pharmacology, important knowledge gaps were evident. Although most reported understanding pharmacokinetics and pharmacodynamics (84.3%) and the variability in efficacy and tolerability (85.1%), fewer expressed confidence in more nuanced areas. About 81% acknowledged the role of inter-individual enzyme variability (e.g., CYP activity), and a smaller proportion (79.6%) indicated they understood how antidepressants are monitored. Notably, awareness of the range of biological samples that could be used for monitoring was limited, with just two-thirds (65.7%) recognizing this aspect. These gaps suggest uneven comprehension, particularly in applied pharmacology and monitoring domains. See Table 2.

### 3.2. Attitudes Toward Prescribing and Monitoring

Attitudes revealed both strengths and areas of concern. While most respondents endorsed the value of follow-up assessments for reducing relapse (88.9%), minimizing disagreements with patients (82.4%), and tailoring treatment (82.4%), there was marked variability on other issues. Fewer than half (44.4%) supported the idea that therapeutic drug monitoring should be standard practice, and only about half (51.0%) endorsed discontinuation of antidepressants in pregnant or lactating women, although a majority (75.9%) preferred dose or drug adjustment in such cases. Over half (53.7%) felt that international guidelines did not fully apply to Jordan’s population, reflecting concerns about contextual relevance. Moreover, 57.0% believed patients view repeated follow-up assessments without dose changes as wasteful, and 71.0% anticipated resistance to dose increases when side effects occur. Social and cultural barriers were also apparent, with only one-third (38.7%) comfortable asking all women of childbearing age about pregnancy intentions. These results point to cautious, sometimes conflicted views that may limit full implementation of evidence-based practices. See Table 3.

### 3.3. Group Comparisons of Knowledge and Attitudes

Analysis revealed significant differences in knowledge and attitude scores across experience levels (*p* < 0.001, η^2^ = 0.17), representing a large effect size. Post hoc Tukey tests indicated that psychiatrists with 11–20 years of experience had significantly higher knowledge scores than those with 0–5 years (*p* = 0.001, Cohen’s d = 0.82) and 6–10 years (*p* = 0.002, d = 0.79). The same group also reported significantly higher attitude scores compared to those with 0–5 years (*p* < 0.001, d = 0.88) and 6–10 years (*p* = 0.001, d = 0.74). Psychiatrists with >20 years of experience also demonstrated higher attitude scores than those with 0–5 years (*p* = 0.038, d = 0.62). No significant pairwise differences were observed beyond these comparisons, and knowledge and attitude scores did not differ significantly by gender (*p* = 0.643 and *p* = 0.607, respectively).

### 3.4. Prescribing Practices and Guideline Adherence

Although nearly all psychiatrists reported adherence to international guidelines when prescribing antidepressants (90.5%), recognition of national Ministry of Health (MOH) guidelines was strikingly low, with only one-third (34.3%) acknowledging them as a standard. Uncertainty was also common, as more than a quarter (26.9%) were unsure whether international recommendations were being followed in practice. This points to limited dissemination or uptake of national standards and potential inconsistencies in guideline-informed care.

Patient safety and minimizing side effects (63.9%) and improving outcomes (61.1%) were the main motivations for following guidelines, yet less than half (43.5%) cited adherence to evidence-based medicine, and only one in five (20.4%) followed guidelines to fulfill regulatory requirements. These findings suggest that prescribing decisions may be driven more by individual judgment than by systematic application of policy or research evidence.

Clinical management also showed areas of variability. While most psychiatrists identified side effects (86.9%) and relapse (69.2%) as reasons for frequent follow-up, fewer consistently considered special populations such as pregnant or breastfeeding women (57.9%), children and adolescents (55.7%), or those with comorbidities like diabetes (42.1%). Follow-up scheduling was inconsistent, with just over half favoring monthly visits (59.4%), while others relied on case-by-case follow-up (20.8%) or only at the time of dose change (11.3%).

Prescribing approaches leaned heavily on experience, with nearly all respondents favoring a “start low and titrate” method (95.2%), but reliance on personal clinical judgment remained high: 40.7% reported “often” using previous experience to guide dosing, and another 21.3% reported doing so “always.” This reliance highlights possible gaps in standardized, guideline-based decision-making.

Laboratory monitoring practices were particularly inconsistent. Only 17.6% of psychiatrists reported always ordering metabolic monitoring, while the majority did so only sometimes (71.3%) and 11.1% never did. Similarly, consultation with internal medicine for patients on multiple medications was mostly ad hoc: while 18.5% always sought collaboration, almost 70% did so only sometimes, and 10.2% limited consultation to cases of adverse effects.

Monitoring by drug class also revealed disparities. While TCAs (67.6%) and MAOIs (58.0%) were most frequently linked with closer monitoring, SSRIs (32.4%) and SNRIs (13.7%) were far less frequently monitored, despite their widespread use in depression treatment.

Taken together, these findings reveal a prescribing landscape characterized by broad endorsement of international guidelines but limited integration of national standards, variable approaches to follow-up, and inconsistent use of laboratory and collaborative monitoring. See Table 4.

No statistically significant associations were found between years of psychiatric experience and key aspects of antidepressant prescribing, including starting dose (*p* = 0.171), reliance on prior clinical experience (*p* = 0.080), preferred frequency of follow-up (*p* = 0.636), frequency of requesting laboratory investigations for metabolic side effects (*p* = 0.053), consulting internal medicine for patients on multiple medications (*p* = 0.103), or the use of international guidelines (*p* = 0.365). In contrast, recognition of Ministry of Health (MOH) guidelines was significantly associated with years of experience (*p* < 0.001), work setting (*p* = 0.038), job position (*p* = 0.027), and number of publications (*p* = 0.001), but not with following international guidelines (*p* = 0.146) or preferred follow-up frequency (*p* = 0.073). Gender was not a significant factor in guideline use (*p* = 0.061), although usage was higher among females (100%) compared with males (87.2%). No associations were observed with years of experience (*p* = 0.371), number of publications (*p* = 0.377), or personal or family psychiatric history (*p* = 1.000). However, job title (*p* = 0.046) and work setting (*p* = 0.002) were significantly associated with guideline use, with junior residents reporting lower use (76%) than senior residents (96%) and consultants (94.2%).

### 3.5. Qualitative Findings: Thematic Analysis of Interviews

Thematic analysis of four semi-structured interviews yielded seven interrelated themes that offer deeper insight into the contextual, systemic, and clinical realities shaping antidepressant prescribing in Jordan. These findings complement the survey data by illustrating how psychiatrists navigate the tension between evidence-based guidelines and the constraints of local practice. The themes, supported by illustrative quotations, are organized into four overarching domains.

#### 3.5.1. Systemic and Structural Constraints

Clinical decision-making under systemic pressures.

Although SSRIs were consistently described as the first-line treatment, psychiatrists noted that actual prescribing was shaped by factors such as insurance coverage, drug availability, and patient comorbidities. As one participant explained: “*Type of insurance plays a big role; within SSRIs we pick what the patient can actually get.*” (Dr3). This reflects how systemic realities often override pharmacological preference, leading to pragmatic rather than purely evidence-driven choices.

Escalation and treatment challenges.

When patients did not respond to first-line treatment, psychiatrists described moving through a sequence of strategies—dose optimization, switching agents, augmentation, or in rare cases, electroconvulsive therapy (ECT). Yet, these options were frequently constrained by systemic barriers such as limited drug availability or resource shortages: “*If no response after 6 weeks at full dose, I either switch or add another agent.*” (Dr4). These accounts underscore the restricted therapeutic toolkit available in practice, despite psychiatrists’ awareness of international treatment algorithms.

#### 3.5.2. Clinical Practice and Monitoring

Monitoring and follow-up practices.

Respondents emphasized the importance of careful follow-up to assess efficacy and side effects, especially early in treatment. Most described seeing patients every 2–4 weeks initially, then monthly once stable: “*I usually see them after 2 weeks, then once a month if things are fine.*” (Dr2). However, they acknowledged that workload pressures, travel distances, and patient resistance sometimes disrupted this schedule. These insights echo survey findings that follow-up is inconsistently implemented, despite widespread recognition of its clinical value.

Therapeutic drug monitoring (TDM).

While psychiatrists were aware of TDM, its use in routine practice was described as rare and largely limited to tricyclic antidepressants (TCAs). One clinician stated: “*I don’t use TDM for SSRIs, maybe for TCAs if absolutely necessary.*” (Dr2). This selective use reflects both systemic barriers, such as resource constraints, and a pragmatic understanding of where monitoring is most clinically useful.

#### 3.5.3. Patient-Level Barriers

Stigma and misconceptions.

A recurring theme was the impact of patient beliefs on adherence. Stigma surrounding psychiatric medications, particularly fears of addiction, frequently led patients to discontinue treatment prematurely: “*Patients fear addiction, so they sometimes stop early.*” (Dr1). This aligns with survey findings where psychiatrists reported significant resistance from patients to dose increases or prolonged follow-up. These patient-level barriers highlight the ongoing need for psychoeducation and culturally sensitive interventions to improve adherence.

#### 3.5.4. Guidelines and Future Directions

Guideline reliance and adaptation.

All interviewees reported following international guidelines such as the DSM and Maudsley, yet views diverged on the relevance of Jordan-specific recommendations. Some stressed the urgent need for local adaptation: “*We really need a Jordan-specific guideline because resources differ here.*” (Dr2). Others felt international standards were sufficient. These perspectives reinforce the survey finding that only one-third of psychiatrists recognized the Ministry of Health guidelines, highlighting both limited dissemination and ambivalence toward national adaptation.

Recommendations for improvement.

Finally, participants proposed concrete steps to improve prescribing practices, including greater dissemination of national guidelines and more structured follow-up systems: “*We need better dissemination of national guidelines and more structured follow-up support.*” (Dr1). These recommendations provide a practitioner-driven roadmap that complements the policy and training reforms suggested in our quantitative analysis.

Taken together, the qualitative themes paint a nuanced picture of psychiatric prescribing in Jordan. Psychiatrists strive to practice evidence-based care, but their decisions are mediated by systemic barriers (insurance, drug availability), patient-level challenges (stigma and misconceptions), and institutional gaps (limited guideline dissemination). These findings enrich the quantitative survey results by revealing the “why” behind the numbers—illustrating how practice patterns are not only shaped by knowledge and attitudes but also by lived constraints within Jordan’s healthcare system.

## 4. Discussion

In this study, we explored the knowledge, attitudes, and practices of psychiatrists in Jordan regarding antidepressant prescription using a mixed-methods approach involving a survey (n = 108) and qualitative interviews (n = 4). Several key findings on guideline adherence, clinical decision-making, and safety practices emerged.

A key finding was the discrepancy in guideline recognition; while the majority of psychiatrists (90.5%) follow international guidelines, less than half (34.3%) recognized Jordan’s national guidelines. This suggests a strong commitment to standardized, evidence-based care but highlights a potential gap in the dissemination and integration of local recommendations. Interviews reinforced this finding: all psychiatrists described relying on international guidelines; like the DSM, Maudsley, and ICD. Others, however, felt international standards were sufficient, highlighting a division in attitudes toward national adaptation. These findings are in line with data from many countries, where heterogeneity is evident regarding adherence to guidelines [18].

A plausible explanation is insufficient exposure to the national guidelines during residency training, a point supported by all the interviewees, who stated they exclusively use international resources. This hypothesis is further supported by our quantitative data, which revealed a statistically significant positive correlation between years of experience and recognition of national guidelines (*p* < 0.001), while no such relationship existed for international guidelines (*p* = 0.146). Furthermore, junior residents reported significantly lower overall guideline use compared to senior residents (76% vs. 96%, *p* = 0.046), reinforcing the idea that exposure and adoption of guidelines intensify as training progresses.

Similar challenges have been reported elsewhere; the effectiveness of guideline dissemination is highly dependent on structured educational strategies during training rather than passive availability [19]. Moreover, it has been evident that without institutional or regional implementation support, national guidelines often fail to achieve widespread adoption [20]. These insights align with our findings and suggest that embedding national guidelines into residency curricula, supported by structured dissemination strategies, may enhance their recognition and use.

Psychiatrists’ motivations for guideline use were primarily patient-centered, most often to manage side effects (86.9%), minimize relapses (69.2%), and ensure safety (63.9%). In contrast, adherence to systemic regulations was among the least-reported motives (20.4%). This highlights a professional drive among Jordanian psychiatrists toward evidence-based practice. However, the low emphasis on regulatory adherence likely reflects the absence of a structural framework to monitor and enforce universal standards of care. Interviews added that patient misconceptions often complicate adherence. Such fears explain why psychiatrists place emphasis on side-effect management and psychoeducation as central to guideline use. This finding is consistent with a study of other Middle Eastern countries, which reported that a lack of administrative structure was a key barrier to evidence-informed policymaking (52.6%) [21].

The majority of respondents (59.4%) reported practicing monthly follow-ups. However, a potential disconnect in patient-doctor communication was evident, with 67.3% acknowledging patients may perceive these follow-ups as an unnecessary financial burden if no changes are made to their treatment, consistent with reported barriers to follow-up in other specialties [22]. Interviews provided further insight: most psychiatrists scheduled visits every 2–4 weeks at initiation, then monthly once stable, but noted that workload and long travel distances often disrupted this schedule. This does not necessarily question the clinical need for regular monitoring but rather points to insufficient communication of its therapeutic purpose, which may be enhanced by providing proper psychoeducation [23]. Of note, we found no statistically significant relationship between follow-up frequency and the psychiatrist’s job role (*p* = 0.636), which may reflect how common these practices are across psychiatric routines.

A strong safety culture was evident, with 95.2% of psychiatrists reporting that they initiate treatment with the lowest effective dose. Despite this, collaboration with internal medicine (IM) specialists, in cases of comorbidities, was inconsistent; 69.4% reported consulting IM only “sometimes”, and 10.2% do so only after adverse effects occur. This limited collaboration is concerning, especially in the absence of dedicated Consultation-Liaison (CL) psychiatry services in Jordan, especially given the importance of structured collaboration between psychiatry and IM to improve diagnosis, treatment outcomes, and patient safety [24].

A significant portion of psychiatrists (52.8%) agreed with using Therapeutic Drug Monitoring (TDM) in specific clinical scenarios like suspected non-adherence or toxicity, which aligns with the literature [25]. However, opinion was divided on making TDM a routine practice, with 44.5% agreeing. This hesitation may be explained by practical barriers, as 56.2% believed that routine TDM would be an additional strain on both the healthcare system and patients. This discrepancy highlights a need for continued medical education to clarify the specific, evidence-based indications for TDM in antidepressant therapy.

When asked for which antidepressant class they would consider ordering TDM, Tricyclic Antidepressants (TCAs) were chosen by 67.6% of respondents, consistent with established guidelines [26]. Interviews confirmed this interpretation: “I don’t use TDM for SSRIs, maybe for TCAs if absolutely necessary.” Notably, 58% also selected Monoamine Oxidase Inhibitors (MAOIs), for which TDM is not generally advised [26]. This unexpected finding could stem from several factors, including a lack of familiarity with MAOIs due to their infrequent use, conflating their significant safety concerns (e.g., risk of serotonin syndrome) with a need for blood level monitoring, or gaps in pharmacological training regarding this specific drug class.

Our analysis demonstrated that clinical experience was significantly associated with psychiatrists’ knowledge and attitudes (*p* < 0.001), consistent with findings from a nationwide survey in Iran showing that greater experience was linked to more positive attitudes and improved knowledge regarding psychotropic use [27]. In contrast, experience did not correlate with self-reported practices in our sample (*p* > 0.05). A Saudi survey similarly reported no significant association between years of practice and lithium prescribing in unadjusted analyses, though their regression suggested lower prescribing among less experienced psychiatrists (*p* = 0.001) [28]. Taken together, these results suggest that clinical practices stabilize early in residency, whereas knowledge and attitudes evolve with continued exposure and experience over time.

International comparisons reveal that the knowledge gaps and variability in antidepressant prescribing observed among Jordanian psychiatrists mirror patterns reported in other regions, albeit with context-specific distinctions. Studies from Europe and North America indicate that while adherence to clinical guidelines for depression treatment is generally higher, barriers such as time constraints, patient non-adherence, and limited awareness of monitoring protocols persist globally [18,26]. In contrast, psychiatrists in low- and middle-income countries (LMICs) such as Iran and Saudi Arabia report challenges comparable to those identified in Jordan, including insufficient local guidelines, resource constraints, and reliance on individual clinical judgment [27,28]. These findings suggest that systemic barriers are not unique to Jordan but are intensified by the structural limitations of LMIC health systems, such as restricted access to therapeutic drug monitoring, limited opportunities for continuing education, and weak coordination between psychiatry and primary care. Strengthening inter-specialty collaboration and developing locally relevant, evidence-informed guidelines could help mitigate these practice gaps in Jordan and similar contexts.

Among these systemic strategies, reforming psychiatric residency training and enforcing policy adherence represent high-impact, feasible starting points. Multi-level intervention is essential to address the gaps in guideline awareness, monitoring, and evidence-based prescribing. Residency training reformation must begin by embedding national guidelines into psychiatric residency curricula through case-based learning, structured workshops, being up-to-date with research objective structured clinical examinations (OSCEs), and board exam questions; strategies proven to enhance knowledge retention and application internationally [29,30], while ensuring trainees remain up-to-date with emerging research [31].

Policy enforcement is critical for sustainability; linking hospital accreditation and continuing medical education (CME) credits to adherence to the national guidelines enhances healthcare practices [32,33]. Guidelines must be updated every 3–5 years, as outlined in the National Mental Health and Substance Use Action Plan 2022–2026 [34]. These suggestions may drive meaningful changes in clinical practice and professional knowledge, fostering a culture where national, evidence-informed standards are routinely applied alongside international benchmarks.

## 5. Limitations

Findings should be considered within the context of some limitations. The cross-sectional design precludes causal inferences, and self-reported data may have been influenced by recall bias or social desirability, potentially leading participants to overstate adherence to guidelines or underreport reliance on personal judgment. Further, although the sample included a substantial proportion of psychiatrists practicing in Jordan, the overall size was modest, and the perspectives of other prescribers of antidepressants, such as family physicians or general practitioners, were not captured.

## 6. Conclusions and Recommendations

Psychiatrists in Jordan demonstrate a general commitment to patient safety and favorable clinical outcomes; however, important gaps remain in the consistent application of evidence-based prescribing practices. Despite widespread adherence to international guidelines, recognition and use of national Ministry of Health (MOH) guidelines were notably limited, underscoring the urgent need for more systematic dissemination and integration of local standards into both residency curricula and routine clinical care. Embedding national guidelines in training and practice would help reduce inconsistencies, strengthen accountability, and ensure care is tailored to the Jordanian context rather than relying solely on international recommendations that may not always be directly applicable.

In parallel, the establishment of a stronger regulatory framework is essential to standardize prescribing practices across sectors, particularly between private clinics, public facilities, and hospital-based care. Such a framework could help close gaps in follow-up practices, laboratory monitoring, and inter-specialty collaboration, which were found to be inconsistent across the sample.

Finally, targeted continuing education and professional development initiatives are needed to address the knowledge and attitude gaps identified, especially among early-career psychiatrists. These should emphasize the rational and evidence-based use of monitoring strategies, cautious management of vulnerable populations such as pregnant women and the elderly, and the importance of systematic follow-up. Without such efforts, prescribing will remain variable and overly reliant on personal judgment rather than structured, guideline-informed practice. Collectively, these measures would help align prescribing practices in Jordan with international standards while also ensuring they are responsive to local needs and systemic realities.

## Figures and Tables

**Table 1:** Participant characteristics (n = 108).

Variable	Frequency	Percentage
**Gender**		
Male	78	72.9%
Female	29	27.1%
**Age**		
Mean (SD)	37.98	(11.68)
**Country of Graduation**		
Jordan	59	62.1%
Iraq	9	9.5%
Algeria	5	5.3%
Egypt	5	5.3%
Ukraine	4	4.2%
Syria	3	3.2%
UK	3	3.2%
China	2	2.1%
Bahrain	1	1.1%
Pakistan	1	1.1%
Russia	1	1.1%
USA	1	1.1%
Yemen	1	1.1%
**Years of Experience**		
0–5 years	55	52.4%
6–10 years	14	13.3%
11–20 years	23	21.9%
>20 years	13	12.4%
Job Position		
Consultant	53	51.0%
Senior Resident	25	24.0%
Junior Resident	26	25.0%
**Work Setting**		
Private clinic	38	36.5%
Public clinic	19	18.3%
Private and Public clinics	8	7.7%
Hospital ward	36	34.6%
Retired	2	1.9%
Military	1	1.0%
**Daily Working Hours**		
<8 h	37	35.2%
8–12 h	64	61.0%
>12 h	4	3.8%
**Patients Seen per Day**		
<10	44	41.9%
11–29	44	41.9%
≥30	17	16.2%
**Monthly Income (JOD)**		
<500	10	9.8%
501–1000	41	40.2%
1001–2000	25	24.5%
2001–3000	17	16.7%
>3000	9	8.8%
**Personal Psychiatric History**		
Yes	24	23.1%
No	80	76.9%
**Family Psychiatric History**		
Yes	23	22.1%
No	81	77.9%
**Number of Publications**		
0	45	43.3%
1–5	43	41.3%
6–10	8	7.7%
>10	8	7.7%

**Table 2:** Descriptive analysis of Knowledge items.

Items	SA (%)	A (%)	N (%)	D (%)	SD (%)	Mean	SD
I understand the pharmacokinetics and pharmacodynamics of different antidepressants.	42 (38.9)	49 (45.4)	13 (12)	2 (1.9)	2 (1.9)	4.18	0.85
I understand the inter-individual variability of clinical efficacy and tolerability of antidepressants.	48 (44.4)	44 (40.7)	10 (9.3)	4 (3.7)	2 (1.9)	4.22	0.9
I understand the inter-individual variability of enzymes (like CYP enzyme) activity.	46 (42.6)	42 (38.9)	11 (10.2)	5 (4.6)	4 (3.7)	4.14	1.02
I understand the way antidepressants are monitored.	38 (35.2)	48 (44.4)	15 (13.9)	4 (3.7)	3 (2.8)	4.06	0.95
There are different biological samples (e.g., blood tests) that could be used to perform therapeutic drug monitoring.	32 (29.6)	39 (36.1)	21 (19.4)	12 (11.1)	4 (3.7)	3.77	1.11

**Table 3:** Descriptive analysis of Attitude items.

Items	SA (%)	A (%)	N (%)	D (%)	SD (%)	Mean	SD
1—Antidepressants may interact with other medications, even those prescribed for non-psychiatric conditions.	50 (46.3)	46 (42.6)	8 (7.4)	1 (0.9)	3 (2.8)	4.29	0.87
2—It is possible to do therapeutic drug monitoring in antidepressant therapy, particularly in cases of suspected noncompliance or intoxication.	12 (11.1)	45 (41.7)	30 (27.8)	19 (17.6)	2 (1.9)	3.43	0.97
3—Therapeutic drug monitoring is recommended when intolerable side effects occur, even at standard doses.	25 (23.1)	37 (34.3)	24 (22.2)	17 (15.7)	5 (4.6)	3.56	1.15
4—Follow-up assessment helps reduce the relapse rate in patients with depression.	57 (52.8)	39 (36.1)	10 (9.3)	1 (0.9)	1 (0.9)	4.39	0.77
5—Follow-up assessment for antidepressants can help minimize disagreements about dosage between doctors and patients.	43 (39.8)	46 (42.6)	13 (12.0)	4 (3.7)	2 (1.9)	4.15	0.91
6—I believe that adding therapeutic drug monitoring to the treatment regimen would cause considerable strain on the healthcare system and patients.	8 (7.6)	51 (48.6)	27 (25.7)	15 (14.3)	4 (3.8)	3.42	0.96
7—Follow-up assessment for antidepressants provides clinicians with documented evidence of tailored treatment adjustments, offering a measure of protection.	43 (39.8)	46 (42.6)	15 (13.9)	3 (2.8)	1 (0.9)	4.18	0.84
8—Follow-up assessment is essential for safety, particularly for drugs with a high risk of overdose.	63 (58.9)	27 (25.2)	12 (11.2)	2 (1.9)	3 (2.8)	4.36	0.95
9—Therapeutic drug monitoring should be a standard practice for all antidepressant prescriptions.	12 (11.1)	36 (33.3)	32 (29.6)	17 (15.7)	11 (10.2)	3.19	1.15
10—Gradual titration of antidepressant doses leads to better outcomes, even if clinical improvement takes longer.	24 (22.2)	65 (60.2)	14 (13.0)	3 (2.8)	2 (1.9)	3.98	0.80
11—I would consider dose adjustment before prescribing different medication in cases of refractory depression.	47 (43.9)	45 (42.1)	10 (9.3)	3 (2.8)	2 (1.9)	4.23	0.87
12—Comorbid conditions affecting pharmacokinetics, such as liver or kidney insufficiency and cardiovascular disease, can influence antidepressant effects in patients.	40 (37.7)	45 (42.5)	17 (16.0)	1 (0.9)	3 (2.8)	4.11	0.90
13—Stopping antidepressants in pregnant or lactating women is important to minimize drug exposure to the fetus or infant.	22 (20.4)	33 (30.6)	20 (18.5)	25 (23.1)	8 (7.4)	3.33	1.25
14—Changing antidepressants type and/or dose in pregnant or lactating women is important to minimize drug exposure to the fetus or infant.	38 (35.2)	44 (40.7)	22 (20.4)	3 (2.8)	1 (0.9)	4.06	0.87
15—Patients with extreme body weights, such as obesity or severe underweight, require special consideration when prescribing antidepressants.	43 (40.2)	47 (43.9)	13 (12.1)	3 (2.8)	1 (0.9)	4.2	0.83
16—When prescribing antidepressants to elderly patients, I consider that they are often on multiple medications and may have a higher sensitivity to side effects.	62 (57.4)	32 (29.6)	11 (10.2)	2 (1.9)	1 (0.9)	4.41	0.82
17—The safety and efficacy standards established for antidepressant use in adults do not apply to children and adolescents.	14 (13.0)	58 (53.7)	16 (14.8)	17 (15.7)	3 (2.8)	3.58	1
18—Patients often face extended waiting times for appointments, leading them to continue taking prescribed doses regardless of their effectiveness.	13 (12.0)	53 (49.1)	27 (25.0)	12 (11.1)	3 (2.8)	3.56	0.94
19—International guidelines may not fully apply to Jordan’s population and therefore I do not follow them strictly.	5 (4.7)	28 (26.2)	23 (21.5)	40 (37.4)	11 (10.3)	2.78	1.09
20—Patients may consider follow-up assessment as a waste of money when its result suggests no change in dose especially when it happens many consecutive times.	11 (10.3)	61 (57.0)	26 (24.3)	8 (7.5)	1 (0.9)	3.68	0.8
21—Patients may resist increasing their dose based on the follow-up assessment results if they start experiencing side effects, even if minimal.	12 (11.2)	76 (71.0)	17 (15.9)	1 (0.9)	1 (0.9)	3.91	0.62
22—It is socially challenging to ask all women of childbearing age about pregnancy intentions before prescribing antidepressants.	7 (6.6)	34 (32.1)	18 (17.0)	38 (35.8)	9 (8.5)	2.92	1.14

**Table 4:** Prescribing practices.

Variable	Frequency	Percentage
**Follow international guidelines?**		
*Yes*	95	90.5%
*No*	10	9.5%
**MOH guideline recognized?**		
*Yes*	37	34.3%
*No*	20	18.5%
*Int. guidelines followed*	22	20.4%
*Not sure*	29	26.9%
**Motivation for guideline following**		
*Patient Safety and minimize side effect*	69	63.9%
*Adhere to EBM*	47	43.5%
*Fulfill regulatory requirements*	22	20.4%
*Improve outcomes*	66	61.1%
**Factors needing frequent follow-up**		
*Side effects*	93	86.9%
*Relapse*	74	69.2%
*Non-responder patient*	65	60.7%
*Hepatic/renal disease*	58	54.2%
*Ischemic heart disease*	51	47.7%
*Extreme weight (obesity/underweight)*	54	50.5%
*Postpartum*	56	52.3%
*Elderly people*	66	61.7%
*Pregnancy/breastfeeding*	62	57.9%
*Smoking*	34	31.8%
*Alcohol use*	54	50.5%
*Controlled substance misuse*	53	40.5%
*Use of another psychotropic drug*	52	48.6%
*Diabetes*	45	42.1%
*Ethnicity*	33	30.8%
*Children and adolescents*	59	55.7%
*IBD or Bowel resection*	44	41.1%
**Antidepressant class used with therapeutic drug monitoring**		
*MAOIs*	58	58.0%
*TCAs*	69	67.6%
*SSRIs*	33	32.4%
*SNRIs*	14	13.7%
*SARIs*	9	8.8%
**Use of previous clinical experience for dosing**		
*Always*	23	21.3%
*Often*	44	40.7%
*Sometimes*	35	32.4%
*Rarely*	5	4.6%
*Never*	1	0.9%
**Frequency of follow-up visits**		
*Only when needed*	22	20.8%
*Dose change*	12	11.3%
*Monthly*	63	59.4%
*Every 3 months*	9	8.5%
*Every 6 months*	0	0%
**Frequency of lab monitoring for metabolic effects**		
*Always*	19	17.6%
*Sometimes*	77	71.3%
*Never*	12	11.1%
Dose initiation method		
*Start low and titrate*	100	95.2%
*Start high*	5	4.8%
**Internal medicine consults for patients on multiple medications.**		
*Always*	20	18.5%
*Sometimes*	75	69.4%
*Never*	2	1.9%
*Only if adverse effects*	11	10.2%

## Data Availability

The dataset is openly available through Figshare at the following link: https://figshare.com/s/6cf09a3b8892149de146 (accessed on 12 September 2025).

## Supplementary Materials

The following supporting information can be downloaded at: https://www.mdpi.com/article/10.3390/healthcare13222954/s1, The full survey instrument and qualitative interview guide used in this study are available to download, File S1: Quantitative Survey and File S2: Interview Guide.

## References

[1] (2022). Global, Regional, and National Burden of 12 Mental Disorders in 204 Countries and Territories, 1990–2019: A Systematic Analysis for the Global Burden of Disease Study 2019. Lancet Psychiatry.

[2] Salameh G., Marais D., Khoury R. (2023). Impact of COVID-19 Pandemic on Mental Health among the Population in Jordan. Int. J. Environ. Res. Public Health.

[3] GBD Results. https://vizhub.healthdata.org/gbd-results.

[4] National Institute for Health and Care Excellence (NICE) (2023). Depression in Adults: Treatment and Management (NG222).

[5] Chu A., Wadhwa R. (2025). Selective Serotonin Reuptake Inhibitors. StatPearls.

[6] Demyttenaere K. (2003). Risk Factors and Predictors of Compliance in Depression. Eur. Neuropsychopharmacol..

[7] Jamison K.R. (2011). An Unquiet Mind: A Memoir of Moods and Madness.

[8] Lee M.-S., Lee H.-Y., Kang S.-G., Yang J., Ahn H., Rhee M., Ko Y.-H., Joe S.-H., Jung I.-K., Kim S.-H. (2010). Variables Influencing Antidepressant Medication Adherence for Treating Outpatients with Depressive Disorders. J. Affect. Disord..

[9] Stahl S.M., Muntner N., Grady M.M. (2021). Stahl’s Essential Psychopharmacology: Neuroscientific Basis and Practical Applications.

[10] Preskorn S.H., Flockhart D.A. (2004). 2010 Guide to Psychiatric Drug Interactions. Prim. Psychiatry.

[11] American Psychiatric Association (2022). Diagnostic and Statistical Manual of Mental Disorders.

[12] Hirsch G.S. (2018). Dosing and Monitoring: Children and Adolescents. Psychopharmacol. Bull..

[13] International Medical Corps Jordan Assessment Report—Understanding the Mental Health and Psycho Social Needs, and Service Utilization of Syrian Refugees and Jordanian Nationals. https://data.unhcr.org/en/documents/details/61303.

[14] World Health Organization (2011). WHO-AIMS Report on Mental Health System in Jordan.

[15] World Health Organization Mental Health Atlas 2020 Country Profile: Jordan. https://www.who.int/publications/m/item/mental-health-atlas-jor-2020-country-profile.

[16] Abdulhaq B., Dardas L.A., Sami O. (2021). Monitoring for the Metabolic Side Effects of Second-generation Antipsychotic Medications: Psychiatrists’ Views and Practices. Perspect. Psychiatr. Care.

[17] Pennazio F., Brasso C., Villari V., Rocca P. (2022). Current Status of Therapeutic Drug Monitoring in Mental Health Treatment: A Review. Pharmaceutics.

[18] Dizet S., Haffen E., Javelot H. (2025). Adherence to Evidence-Based Treatment Guidelines for Depression: A Systematic Review. BMC Psychiatry.

[19] Koenig E., Hoffmann U., Fegert J.M., Keller F., Sicorello M., Spohrs J., Kraus L., Nickel S., Schmahl C., Abler B. (2024). Training Approaches for the Dissemination of Clinical Guidelines for NSSI: A Quasi-Experimental Trial. Child Adolesc. Psychiatry Ment. Health.

[20] Pereira V.C., Silva S.N., Carvalho V.K.S., Zanghelini F., Barreto J.O.M. (2022). Strategies for the Implementation of Clinical Practice Guidelines in Public Health: An Overview of Systematic Reviews. Health Res. Policy Syst..

[21] El-Jardali F., Lavis J.N., Ataya N., Jamal D., Ammar W., Raouf S. (2012). Use of Health Systems Evidence by Policymakers in Eastern Mediterranean Countries: Views, Practices, and Contextual Influences. BMC Health Serv. Res..

[22] Bani Salameh R., Al-Smadi A., Gammoh O., Shajrawi A., Ashour A., Alrfooh O., Fitzsimons D., Hatahet T., Binsaleh A.Y., Shilbayeh S.A.R. (2025). Exploring the Implemented Guidelines for Dyslipidemia Treatment and Care among Nurses and Physicians: A Qualitative Study in Jordan. PLoS ONE.

[23] Noguchi H., Tarutani S., Takei Y., Matsumoto K., Okamura T., Yoneda H. (2025). Prognostic Impact of Psychoeducation Program Completion on Inpatients with Schizophrenia: A Pilot Cohort Study. BMC Psychiatry.

[24] Țenea-Cojan Ș.-T., Dinescu V.-C., Gheorman V., Dragne I.-G., Gheorman V., Forțofoiu M.-C., Fortofoiu M., Dobrinescu A.G. (2025). Exploring Multidisciplinary Approaches to Comorbid Psychiatric and Medical Disorders: A Scoping Review. Life.

[25] Fiaturi N., Greenblatt D.J., Macaluso M., Preskorn S.H. (2018). Therapeutic Drug Monitoring of Antidepressants. Antidepressants.

[26] Hiemke C., Bergemann N., Clement H., Conca A., Deckert J., Domschke K., Eckermann G., Egberts K., Gerlach M., Greiner C. (2018). Consensus Guidelines for Therapeutic Drug Monitoring in Neuropsychopharmacology: Update 2017. Pharmacopsychiatry.

[27] Rezaie L., Nazari A., Safari-Faramani R., Shohaimi S., Khazaie H. (2022). Iranian Psychiatrists’ Attitude towards Clozapine Use for Patients with Treatment-Resistant Schizophrenia: A Nationwide Survey. BMC Psychiatry.

[28] Alolayan M.A., Almadani A.H., Alrashed G.K., Alothaim J., Hussein W. (2025). Attitudes toward Lithium Prescription among Psychiatrists in Saudi Arabia: A Cross-Sectional Study. Front. Psychiatry.

[29] Kumaravel B., Stewart C., Ilic D. (2021). Development and Evaluation of a Spiral Model of Assessing EBM Competency Using OSCEs in Undergraduate Medical Education. BMC Med. Educ..

[30] McLean S.F. (2016). Case-Based Learning and Its Application in Medical and Health-Care Fields: A Review of Worldwide Literature. J. Med. Educ. Curric. Dev..

[31] Munk-Jørgensen P., Blanner Kristiansen C., Uwawke R., Larsen J.I., Okkels N., Christiansen B., Hjorth P. (2015). The Gap between Available Knowledge and Its Use in Clinical Psychiatry. Acta Psychiatr. Scand..

[32] Gupta K., Mandlik D., Patel P., Patel D., Patel K.D. (2019). Does Continuing Medical Education (CME) Activity Contribute to Learning Gain: An Objective Evaluation. Indian J. Otolaryngol. Head Neck Surg..

[33] Alhawajreh M.J., Paterson A.S., Jackson W.J. (2023). Impact of Hospital Accreditation on Quality Improvement in Healthcare: A Systematic Review. PLoS ONE.

[34] National Mental Health and Substance Use Action Plan 2022-2026. https://www.moh.gov.jo/ebv4.0/root_storage/en/eb_list_page/national_mhsu_action_plan_2022-2026_(english)_(2)-0.pdf.

