# Supplementary material for: Knowledge Gaps and Systemic Challenges in Antidepressant Prescribing: Insights from Jordanian Psychiatry Practice
Source: Healthcare (Basel). 2025 Nov 18;13(22):2954. doi: 10.3390/healthcare13222954 (PMC12652151; doi:10.3390/healthcare13222954)
Supplement: Supplementary file 1 [file healthcare-13-02954-s001.zip › File S1 Quantitative Survey.pdf]

Assessment of Knowledge on Antidepressant Prescription

This section evaluates your knowledge about antidepressant prescriptions. It contains five statements. Please rate how much you agree with each of them.

1. **I understand the pharmacokinetics and pharmacodynamics of different antidepressants.**
   - ☐ Strongly Disagree
   - ☐ Disagree
   - ☐ Neutral
   - ☐ Agree
   - ☐ Strongly Agree
2. **I understand the inter-individual variability of clinical efficacy and tolerability of antidepressants.**
   - ☐ Strongly Disagree
   - ☐ Disagree
   - ☐ Neutral
   - ☐ Agree
   - ☐ Strongly Agree
3. **I understand the inter-individual variability of enzymes (like CYP enzyme) activity.**
   - ☐ Strongly Disagree
   - ☐ Disagree
   - ☐ Neutral
   - ☐ Agree
   - ☐ Strongly Agree
4. **I understand the way antidepressants are monitored.**
   - ☐ Strongly Disagree
   - ☐ Disagree
   - ☐ Neutral
   - ☐ Agree
   - ☐ Strongly Agree
5. **There are different biological samples (e.g., blood tests) that could be used to perform therapeutic drug monitoring.**
   - ☐ Strongly Disagree
   - ☐ Disagree
   - ☐ Neutral
   - ☐ Agree
   - ☐ Strongly Agree

Assessment of Attitudes Toward Antidepressant Prescription

This section explores your attitudes and perspectives toward antidepressant prescriptions. It contains 22 statements. Please rate how much you agree with each of them.

1. **Antidepressants may interact with other medications, even those prescribed for non-psychiatric conditions.**
   - ☐ Strongly Disagree
   - ☐ Disagree
   - ☐ Neutral
   - ☐ Agree
   - ☐ Strongly Agree
2. **It is possible to do therapeutic drug monitoring in antidepressant therapy, particularly in cases of suspected noncompliance or intoxication.**
   - ☐ Strongly Disagree
   - ☐ Disagree
   - ☐ Neutral
   - ☐ Agree
   - ☐ Strongly Agree
3. **Therapeutic drug monitoring is recommended when intolerable side effects occur, even at standard doses.**
   - ☐ Strongly Disagree
   - ☐ Disagree
   - ☐ Neutral
   - ☐ Agree
   - ☐ Strongly Agree
4. **Follow-up assessment helps reduce the relapse rate in patients with depression.**
   - ☐ **Strongly Disagree**
   - ☐ **Disagree**
   - ☐ **Neutral**
   - ☐ **Agree**
   - ☐ **Strongly Agree**
5. **Follow-up assessment for antidepressants can help minimize disagreements about dosage between doctors and patients.**
   - ☐ Strongly Disagree
   - ☐ Disagree
   - ☐ Neutral
   - ☐ Agree
   - ☐ Strongly Agree
6. **I believe that adding therapeutic drug monitoring to the treatment regimen would cause considerable strain on the healthcare system and patients.**
   - ☐ Strongly Disagree
   - ☐ Disagree

☐ Neutral

☐ Agree

- ☐ Strongly Agree

7. **Follow-up assessment for antidepressants provides clinicians with documented evidence of tailored treatment adjustments, offering a measure of protection.**
   - ☐ Strongly Disagree
   - ☐ Disagree
   - ☐ Neutral
   - ☐ Agree
   - ☐ Strongly Agree
8. **Follow-up assessment is essential for safety, particularly for drugs with a high risk of overdose.**
   - ☐ Strongly Disagree
   - ☐ Disagree
   - ☐ Neutral
   - ☐ Agree
   - ☐ Strongly Agree
9. **Therapeutic drug monitoring should be a standard practice for all antidepressant prescriptions.**
   - ☐ Strongly Disagree
   - ☐ Disagree
   - ☐ Neutral
   - ☐ Agree
   - ☐ Strongly Agree
10. **Gradual titration of antidepressant doses leads to better outcomes, even if clinical improvement takes longer.**
    - ☐ Strongly Disagree
    - ☐ Disagree
    - ☐ Neutral
    - ☐ Agree
    - ☐ Strongly Agree
11. **I would consider dose adjustment before prescribing different medication in cases of refractory depression.**
    - ☐ Strongly Disagree
    - ☐ Disagree
    - ☐ Neutral
    - ☐ Agree
    - ☐ Strongly Agree
12. **Comorbid conditions affecting pharmacokinetics, such as liver or kidney insufficiency and cardiovascular disease, can influence antidepressant effects in patients.**
    - ☐ Strongly Disagree
    - ☐ Disagree
    - ☐ Neutral

☐ Agree

- ☐ Strongly Agree

13. Stopping antidepressants in pregnant or lactating women is important to minimize drug exposure to the fetus or infant.

- ☐ Strongly Disagree
- ☐ Disagree
- ☐ Neutral
- ☐ Agree
- ☐ Strongly Agree

14. Changing antidepressant type and/or dose in pregnant or lactating women is important to minimize drug exposure to the fetus or infant.

- ☐ Strongly Disagree
- ☐ Disagree
- ☐ Neutral
- ☐ Agree
- ☐ Strongly Agree

15. Patients with extreme body weights, such as obesity or severe underweight, require special consideration when prescribing antidepressants.

- ☐ Strongly Disagree
- ☐ Disagree
- ☐ Neutral
- ☐ Agree
- ☐ Strongly Agree

16. When prescribing antidepressants to elderly patients, I consider that they are often on multiple medications and may have a higher sensitivity to side effects.

- ☐ Strongly Disagree
- ☐ Disagree
- ☐ Neutral
- ☐ Agree
- ☐ Strongly Agree

17. The safety and efficacy standards established for antidepressant use in adults do not apply to children and adolescents.

- ☐ Strongly Disagree
- ☐ Disagree
- ☐ Neutral
- ☐ Agree
- ☐ Strongly Agree

18. Patients often face extended waiting times for appointments, leading them to continue taking prescribed doses regardless of their effectiveness.

- ☐ Strongly Disagree
- ☐ Disagree
- ☐ Neutral

☐ Agree
☐ Strongly Agree

19. **International guidelines may not fully apply to Jordan's population and therefore I do not follow them strictly.**
   - ☐ Strongly Disagree
   - ☐ Disagree
   - ☐ Neutral
   - ☐ Agree
   - ☐ Strongly Agree
20. **Patients may consider follow-up assessment as a waste of money when its result suggests no change in dose, especially when it happens many consecutive times.**
   - ☐ Strongly Disagree
   - ☐ Disagree
   - ☐ Neutral
   - ☐ Agree
   - ☐ Strongly Agree
21. **Patients may resist increasing their dose based on the follow-up assessment results if they started experiencing side effects, even if minimal.**
   - ☐ Strongly Disagree
   - ☐ Disagree
   - ☐ Neutral
   - ☐ Agree
   - ☐ Strongly Agree
22. **It is socially challenging to ask all women of childbearing age about pregnancy intentions before prescribing antidepressants.**
   - ☐ Strongly Disagree
   - ☐ Disagree
   - ☐ Neutral
   - ☐ Agree
   - ☐ Strongly Agree

Assessment of Practices in Antidepressant Prescriptions

This section focuses on evaluating your current practices related to Antidepressant Prescriptions.

1. **Do you follow any international guidelines when prescribing antidepressant doses?**
   - ☐ Yes

☐ No

2. **Are there any guidelines established by the Ministry of Health in Jordan that are recognized as the national standard?**
   - ☐ Yes, there are recognized national guidelines.
   - ☐ No, there are no recognized national guidelines.
   - ☐ No, International guidelines are followed.
   - ☐ I am not sure.
3. **If you follow any national or international guidelines for antidepressant prescriptions, what motivates you to do so?**
   - ☐ To ensure patient's safety and minimize side effects.
   - ☐ To adhere to evidence-based medicine.
   - ☐ To fulfill institutional or regulatory requirements.
   - ☐ To improve treatment outcomes and patient's satisfaction.
4. **Which of the following warrants frequent follow-up assessment or antidepressants?**
   - ☐ Side effects.
   - ☐ Relapse.
   - ☐ Non-responder patient.
   - ☐ Hepatic, renal disease.
   - ☐ Ischemic heart disease.
   - ☐ Extreme weight on both ends.
   - ☐ Postpartum.
   - ☐ Elderly people.
   - ☐ Pregnancy/breastfeeding.
   - ☐ Smoking.
   - ☐ Alcohol use.
   - ☐ Controlled substance misuse/abuse/dependence.
   - ☐ Use of another psychotropic drug simultaneously with antidepressants.
   - ☐ Diabetes.
   - ☐ Ethnicity.
   - ☐ Children and adolescents.
   - ☐ Inflammatory bowel diseases or bowel resection.
5. **Which antidepressants class would you use therapeutic drug monitoring for if applicable?**
   - ☐ Monoamine oxidase inhibitors (MAOIs)
   - ☐ Tricyclic antidepressants (TCAs)
   - ☐ Selective serotonin reuptake inhibitors (SSRIs)
   - ☐ Serotonin-noradrenaline reuptake inhibitors (SNRIs)
   - ☐ Serotonin antagonists and reuptake inhibitors (SARIs)
   - ☐ Others

If others, Please Specify:____

6. **How often do you use your previous clinical experience with similar patients to guide decisions about antidepressant dosages?**
   - ☐ Always
   - ☐ Often
   - ☐ Sometimes
   - ☐ Rarely
   - ☐ Never
7. **How often do you believe follow-up assessment visits should be requested?**
   - ☐ Only when clinically indicated
   - ☐ With each dose change
   - ☐ Monthly
   - ☐ Every 3 months
   - ☐ Every 6 months
8. **How often do you request laboratory results to monitor metabolic antidepressant side effects?**
   - ☐ Always
   - ☐ Sometimes
   - ☐ Never
9. **What dose do you usually start your patients on?**
   - ☐ I start with the smallest dose and gradually increase it until the patient reaches the effective dose.
   - ☐ I start immediately with a large dose, so we gain the effect sooner.
10. **How often do you consult internal medicine with patients on multiple medications?**
    - ☐ Always
    - ☐ Sometimes
    - ☐ Never
    - ☐ Only if the patient complains of any adverse effects.

Demographic Information

This section collects demographic details to help categorize and analyze the responses. All information provided will remain confidential.

1. **What is your gender?**
   - ☐ Male
   - ☐ Female
2. **What is your age?**

   —------------------ years old

3. **What is the country of your graduation?**

—---

4. **Where are you working?**
   - ☐ Private clinic
   - ☐ Public clinic
   - ☐ Private and Public clinics
   - ☐ Hospital ward
   - ☐ Other:
5. **What is the number of years of experience in psychiatry?**
   - ☐ 0-5
   - ☐ 6-10
   - ☐ 11-20
   - ☐ >20
6. **What is your job position?**
   - ☐ Consultant
   - ☐ Senior trainee (resident)
   - ☐ Junior trainee (resident)
7. **How many hours do you work in a day?**
   - ☐ <8
   - ☐ 8-12
   - ☐ >12
8. **On average, how many patients do you see each day?**
   - ☐ <10
   - ☐ 11-29
   - ☐ >30
9. **What is your average monthly income?**
   - ☐ <500
   - ☐ 501-1000
   - ☐ 1001-2000
   - ☐ 2001-3000
   - ☐ 3000
10. **Have you ever experienced psychiatric illnesses or disorders?**
    - ☐ Yes
    - ☐ No
11. **Is there any family history of psychiatric illness?**
    - ☐ Yes
    - ☐ No
12. **How many scientific papers have you published?**
    - ☐ 0
    - ☐ 1-5

☐ 6-10

☐ >10
