# Supplementary material for: Knowledge Gaps and Systemic Challenges in Antidepressant Prescribing: Insights from Jordanian Psychiatry Practice
Source: Healthcare (Basel). 2025 Nov 18;13(22):2954. doi: 10.3390/healthcare13222954 (PMC12652151; doi:10.3390/healthcare13222954)
Supplement: Supplementary file 1 [file healthcare-13-02954-s001.zip › File S2 Interview Guide.pdf]

Interview Guide: Antidepressant Prescription Practices

1. Introduction and Consent

Thank you for agreeing to participate in this interview. This study aims to understand antidepressant prescription practices among psychiatrists in Jordan. Your input is invaluable to this research, and all responses will remain anonymous and confidential. You may skip any question or stop the interview at any point.
Are you comfortable proceeding?

2. General Prescription Practices

- Can you describe your general approach to prescribing antidepressants?

- What factors influence your choice of a specific antidepressant?

- How do you monitor the effectiveness and side effects of antidepressants in your patients?

- Do you use clinical judgment, follow-up visits, lab tests, or a combination of these?

- How do patient-specific factors (e.g., comorbidities, age, weight, socioeconomic status) influence your antidepressant prescription decisions?

- How often do you need to adjust doses or switch medications for patients with refractory depression?

3. Therapeutic Drug Monitoring (TDM)

- What is your opinion on using therapeutic drug monitoring (TDM) in antidepressant therapy?

- Do you currently use TDM? If yes, in what circumstances? If not, why not?

- In what clinical situations do you believe TDM would be most beneficial (e.g., noncompliance, toxicity, treatment resistance)?

4. Follow-Up and Monitoring Practices

- How often do you schedule follow-up assessments for patients on antidepressants?

- What factors influence the frequency of these follow-ups?

- Do you believe follow-up visits effectively improve patient outcomes? Why or why not?

- What are the main barriers to regular follow-up visits (e.g., patient resistance, scheduling, healthcare system issues)?

- How do patients typically respond to dose adjustments or medication changes during follow-up visits?

- How do you manage patients who stop taking antidepressants without consultation?

5. Challenges in Practice

- What are the most significant challenges you face when prescribing antidepressants?

- Are these challenges patient-related, system-related, or guideline-related?

- How do cultural or social factors (e.g., stigma, family influence) affect patients' adherence to antidepressant therapy?

- How do financial constraints influence patient access to antidepressants or monitoring?

- How do you handle antidepressant prescriptions for special populations:
- Pregnant or lactating women?
- Elderly patients with comorbidities?
- Children and adolescents?

6. Attitudes Toward Guidelines

- Are you aware of any national guidelines for antidepressant prescriptions in Jordan?

- Do you think there is a need for standardized national guidelines?

- Do you follow international guidelines when prescribing antidepressants?

- What challenges do you face in applying these guidelines in Jordan's clinical context?

- How well do you think current international guidelines address the needs of Jordanian patients?

- What would you like to see in locally adapted antidepressant prescribing guidelines?

7. Patient-Centered Challenges

- What misconceptions or beliefs do patients in Jordan commonly have about antidepressant therapy?

- How do you address these misconceptions during consultations?

- How do you deal with patients who are resistant to dose adjustments or medication changes, especially due to side effects?

- What strategies do you use to improve medication adherence among your patients?

- Do you notice any specific patterns regarding antidepressant adherence in special populations (e.g., elderly, postpartum women, adolescents)?

8. Systemic and Resource-Related Issues (??)

- What systemic barriers do you face in prescribing and monitoring antidepressants (e.g., lack of lab facilities, time constraints, healthcare system resources)?

- How accessible are therapeutic drug monitoring tools or tests in your practice?

- What improvements in the healthcare system do you think would help you prescribe and monitor antidepressants more effectively?

9. Recommendations and Final Reflections

- What changes or improvements would you recommend to enhance antidepressant prescription practices in Jordan?

- In your opinion, what would be the most significant factor in improving patient outcomes with antidepressants?

- Is there anything else you would like to share about your experience with antidepressant prescription practices?

10. Closing Statement

Thank you for your valuable insights and time. Your responses will greatly contribute to understanding and improving antidepressant prescription practices in Jordan. If you have any additional thoughts later, please feel free to reach out to our team.
